# Rare Complication Post-Conization for Cervical Dysplasia: Rectovaginal Fistula

**DOI:** 10.3390/clinpract13050091

**Published:** 2023-08-24

**Authors:** Paolo Meloni, Sara Izzo, Claudia De Intinis, Terenzia Simari, Mariangela Motzo, Riccardo Picazzo, Rodolfo Brizio, Cristina Vignale, Marcello Molle, Luciano Izzo, Paolo Izzo

**Affiliations:** 1 SC Ostetricia Ginecologia, Ospedale Imperia, ASL1 Imperiese, 18038 Sanremo, Italy; meloni.paolo@libero.it (P.M.); m.motzo@asl1.liguria.it (M.M.); 2 Plastic Surgery Unit, Multidisciplinary Department of Medical-Surgical and Dental Specialties, Università degli Studi della Campania “Luigi Vanvitelli”, Piazza Luigi Miraglia, 80138 Naples, Italy; sa_izzo@hotmail.it (S.I.); marcello.molle@unicampania.it (M.M.); 3 “Pietro Valdoni” Department of Surgery, Policlinico “Umberto I”, “Sapienza” University of Rome, viale del Policlinico 155, 00161 Rome, Italy; deintinis.1891513@studenti.uniroma1.it (C.D.I.); p_izzo@hotmail.it (L.I.); 4 Ambulatorio Specialistico Ginecologia Ostetricia, ASL1 Imperiese, 18038 Sanremo, Italy; terenziasimari@tiscali.it; 5 SC Radiologia, ASL1 Imperiese, 18038 Sanremo, Italy; r.picazzo@asl1.liguria.it; 6 SC Anatomia Patologica, ASL1 Imperiese, 18038 Sanremo, Italy; r.brizio@asl1.liguria.it (R.B.); c.vignale@asl1.liguria.it (C.V.)

**Keywords:** cervical dysplasia, rectovaginal fistula, conization, complication post-conization

## Abstract

(1) Background: High-grade cervical dysplasia is primarily caused by human papillomavirus (HPV) infection. Conservative surgery is the preferred treatment approach for this condition. The most commonly employed technique is the loop electrosurgical excision procedure (LEEP), which involves removing the affected portion of the cervix. Excisional treatments are often curative, and complications are typically rare and minor. (2) Methods: The loop electrosurgical excision procedure (LEEP) is the standard method used for conservative surgery in high-grade cervical dysplasia. It entails the excision of the specific area of the cervix where the abnormal cells are present. The procedure employs a wire loop carrying an electrical current to remove the affected tissue. (3) Results: Excisional treatments, such as LEEP, have shown to be effective in treating high-grade cervical dysplasia. They have a high success rate in eliminating abnormal cells and reducing the risk of cervical cancer. Complications associated with LEEP are infrequent and usually minor. Short-term complications may include bleeding, which can be managed easily. Long-term complications may involve cervical canal stenosis, which can impact fertility. (4) Conclusions: Conservative surgery, particularly the loop electrosurgical excision procedure (LEEP), is the preferred and effective treatment for high-grade cervical dysplasia caused by HPV infection. It offers a high cure rate with rare and minor complications. While short-term bleeding is a common occurrence, it is manageable. Long-term complications such as cervical canal stenosis may impact fertility. However, an extremely rare and possibly unique complication described in this case is the development of a vaginorectal fistula. This complication is likely due to indirect thermal injury resulting from compromised tissue. Further research is needed to better understand and prevent such complications.

## 1. Introduction

The current morphological criteria for histological diagnosis of cervical lesions are based on the WHO 2014 classification [1]. Within the context of high-grade squamous intraepithelial lesion (HSIL), a distinction is necessary between moderate-grade lesions (moderate dysplasia/CIN2) and more severe lesions (severe dysplasia, carcinoma in situ/CIN3) [2,3]. The recommended treatment is excisional treatment, specifically conization, in which the pathological tissue is removed for subsequent histological examination. Conization allows for the removal of all visible pathological tissue observed during colposcopy and serves both diagnostic and therapeutic purposes. The excised tissue includes the exocervix and endocervix and is shaped like a cone/cylinder with an endocervical apex. Various techniques can be employed for the excision, such as using a cold knife, laser, loop electrosurgical excision procedure (LEEP), and large-loop excision of the transformation zone (LLETZ) [4,5]. Currently, generators capable of emitting high-frequency alternating currents (400 KHz–4 MHz) at low voltage are used, with variable power ranging from 100 to 400 W. The active electrodes employed are loop-shaped and can have different configurations (circular, rectangular, square) with diameters and depths ranging from 0.5 to 2 cm [6].

The diameter of the wire, made of steel and tungsten, does not exceed 0.2 mm. This characteristic allows for a high density of current to pass through it, resulting in the vaporization of the tissue proximally to the wire due to the high temperature produced on a very small surface area. The loop is slowly inserted into the cervix to achieve the excision of a roughly conical-shaped sample [7]. Immediate complications may include intraoperative bleeding (4.2–7.9%) and postoperative bleeding (0.4–2.6%), postoperative infections (0.3–3.5%), and damage to adjacent organs (0.06–0.5%) [8,9]. Late complications include cervical canal stenosis, which can cause dysmenorrhea and hematometra in fertile patients, or it can mask uterine bleeding in postmenopausal women, which can be an early symptom of endometrial neoplasia [10,11]. The excisional techniques appear to slightly worsen the obstetric outcome in subsequent pregnancies [12,13]. Currently, it is not known whether the treatment can cause future fertility problems or affect the course of subsequent pregnancies. Some studies have reported a modestly increased risk of miscarriage and preterm delivery directly proportional to the size of the excised cone [14].

## 2. Case Report

A 34-year-old woman underwent a screening Pap test with an H-SIL result, which prompted a counseling session regarding the significance of the high-grade lesion and further diagnostic tests. She underwent a diagnostic colposcopy with biopsy.

**Figure 1:**
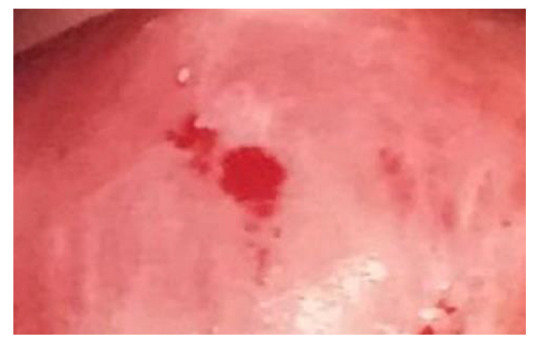
Thin white epithelium after application of acetic acid: a whitish area on the upper lip of the cervix above the external uterine orifice (OUE) can be observed. The whitish color distinguishes it from the pinkish color of the remaining healthy portio. The biopsy is performed under colposcopic guidance at 12 o’clock, exactly in the middle of the region characterized by the whitish mucosal reaction after the application of 5% acetic acid.

**Figure 2:**
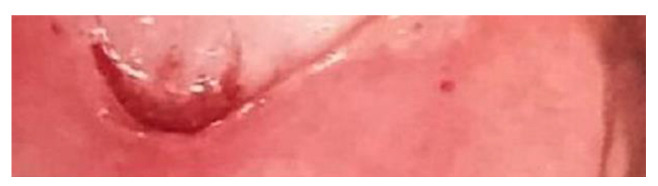
Lugol application: the portio appears brown in the physiological component. The pathological area does not pick up the solution and therefore appears yellowish-reddish, exactly in the region that appeared whitish after the application of acetic acid. This second staining confirms the anatomical region to be biopsied.

Colposcopy is the in-depth examination used in the second level of screening for the prevention of cervical cancer to investigate a positive screening test result (Pap test or HPV test), i.e., to confirm the presence or absence of pre-cancerous or cancerous lesions of the cervix. To perform such an examination, it is necessary to use a colposcope, an optical instrument equipped with a light source and with magnifications varying from 6 to 40 times, with optics between 200 and 250 mm. Colposcopic examination has a sensitivity of about 90% and a specificity of about 85%. Its limitation is endocervical lesions with the squamo-columnar junction (GSC), which is visible at fertile age in about 80–90% of cases and becomes barely visible in post-menopause [15,16,17,18]. Observation of the cervix proceeds in three steps: the first step is to assess the cervix after cleansing with saline solution; the second step is to assess after cleansing with 3 or 5% acetic acid solution (Figure 1); the last step is to assess after application of Lugol’s iodine–iodine solution for observation of the Schiller test (Figure 2).

The application of the described solutions helps to further differentiate the cervical lesion, indicating where to perform the targeted biopsy.

The histological result showed CIN 2, and a proposal for a conization procedure was made. Counseling regarding the procedure was provided, and informed consent was obtained. The doctor who performed the operation is an experienced surgeon who has performed about two hundred conjectures. The appropriate treatment was excisional through a single passage of a fragment of cervical tissue; the aim was the complete excision of the dysplastic lesion and thus obtaining an operative piece with lesion-free margins.

**Figure 3:**
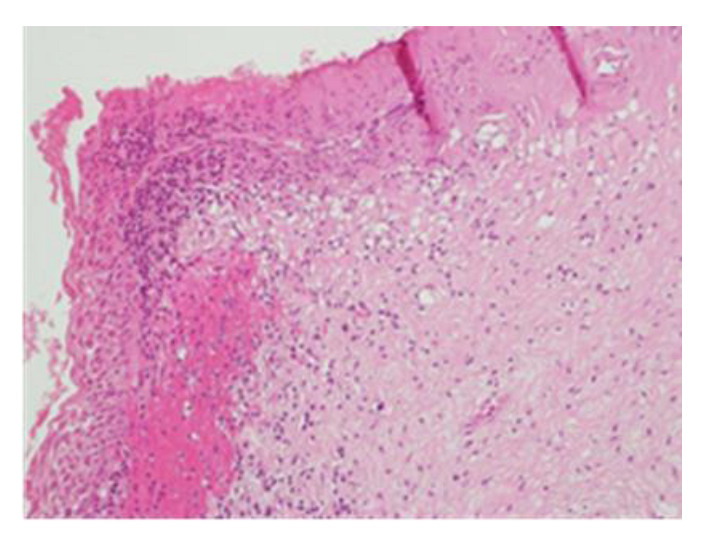
Histological sample of the excised tissue representing the main portion of the excised cervix. Morphologically, enlarged and hyperchromatic nuclei, clumped chromatin, irregularities, notches in the nuclear membrane, and an altered nuclear/cytoplasmic ratio can be observed. The lesion appears extensive at the electroresorptive margins. The lesion is compatible with a diagnosis of CIN3, with an aggravation of the initial CIN2 pathology.

After preparing the surgical field, vaginal valves were placed, and colposcopy was performed in the operating room, revealing a cervical lesion that was larger than initially diagnosed. The margins were delineated, and the cone was excised using a diathermic loop. The diathermic loop used was a half-round ring-shaped end electrode with dimensions of 22 mm × 12 mm and a length of 13 cm. Subsequently, enlargements were necessary to ensure the complete removal of the macroscopically visible lesion. During the surgical procedures, there was significant bleeding, likely originating from the right cervical branch of the uterine artery. Hemostatic stitches were applied to the right vaginal wall to control the bleeding. The application of the stitches was successful in achieving hemostasis, as superficial stitches would have resulted in failure to contain the bleeding with potentially severe consequences for the patient. The ureter was not injured during the placement of the stitches. The histological examination classified the lesion as CIN3, which is internationally classified as carcinoma in situ. This lesion was present in all the margins excised following the cone procedure (Figure 3).

**Figure 4:**
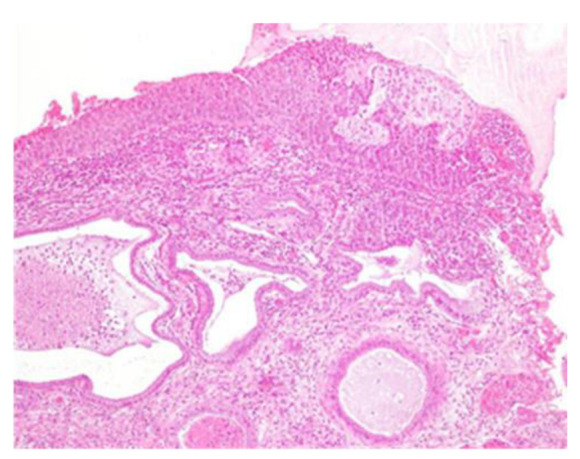
CIN3 histological sample of the left enlargement margin; also, in this case, enlarged and hyperchromatic nuclei, clumped chromatin, irregularities and indentations in the nuclear membrane, and an altered nuclear/cytoplasmic ratio are observed.

**Figure 5:**
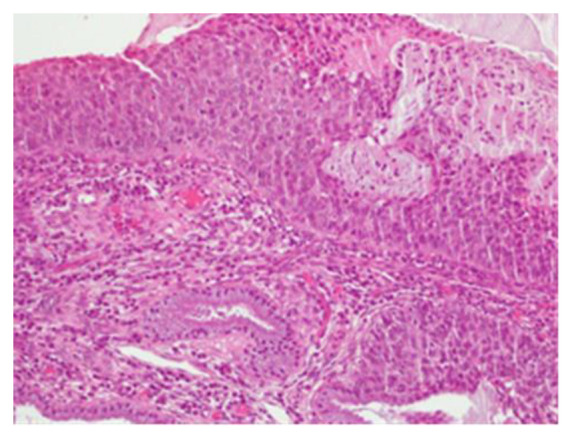
CIN3 histological sample of the right widening margin. In this section, there are also the histological features compatible with CIN 3.

Mitotic figures at various levels are also frequent (Figure 4 and Figure 5).

**Figure 6:**
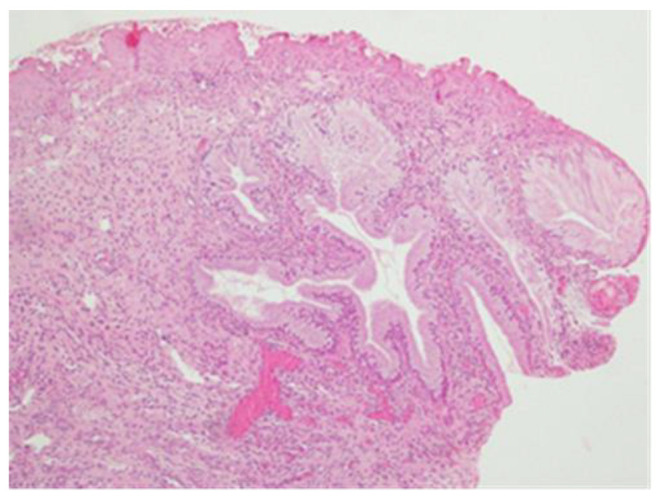
No identifiable lesion in the residual cervix canal.

The lesion was completely removed in the small cone procedure performed on the cervical canal; therefore, in the portion of the cervix that was not removed, no histological lesion is present (Figure 6).

In the postoperative course, the patient experienced nonspecific symptoms: she remained afebrile, had mild pelvic pain at the site of the vaginal suture, which was managed with paracetamol as needed, and had diarrhea throughout the hospital stay.

The patient was examined and there were regular surgical findings at the level of the portio and no vaginal discharge. The pain symptoms improved, but the dysentery did not.

Only on the fifth postoperative day were malodorous secretions observed in the vagina, and the patient was promptly examined for suspected vagino-rectal fistula. Immediate antibiotic therapy was initiated.

Radiographic examination clearly demonstrated free abdominal air, with significant subdiaphragmatic gas collections. The finding was confirmed through CT examination, which also demonstrated diffuse inflammatory involvement of the soft tissues of the pelvis, particularly near the recto-uterine structures. The presence of small gas bubbles in the recto-vaginal space led to the diagnosis of vaginal fistula-rectum. Given the urgency, for a better definition of the findings, it was decided to proceed with a barium enema in order to quickly verify the presence of the fistulous pathway. The examination was conducted by introducing gastrografin into the vagina. The contrast medium partly flowed into the rectum, confirming the diagnosis of recto-vaginal fistula.

**Figure 7:**
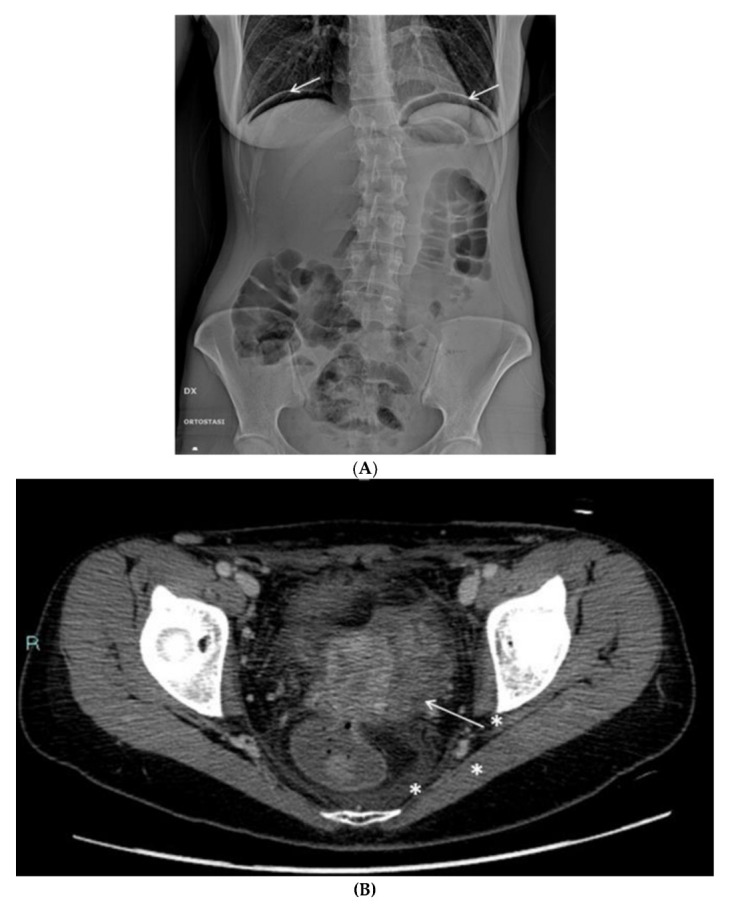

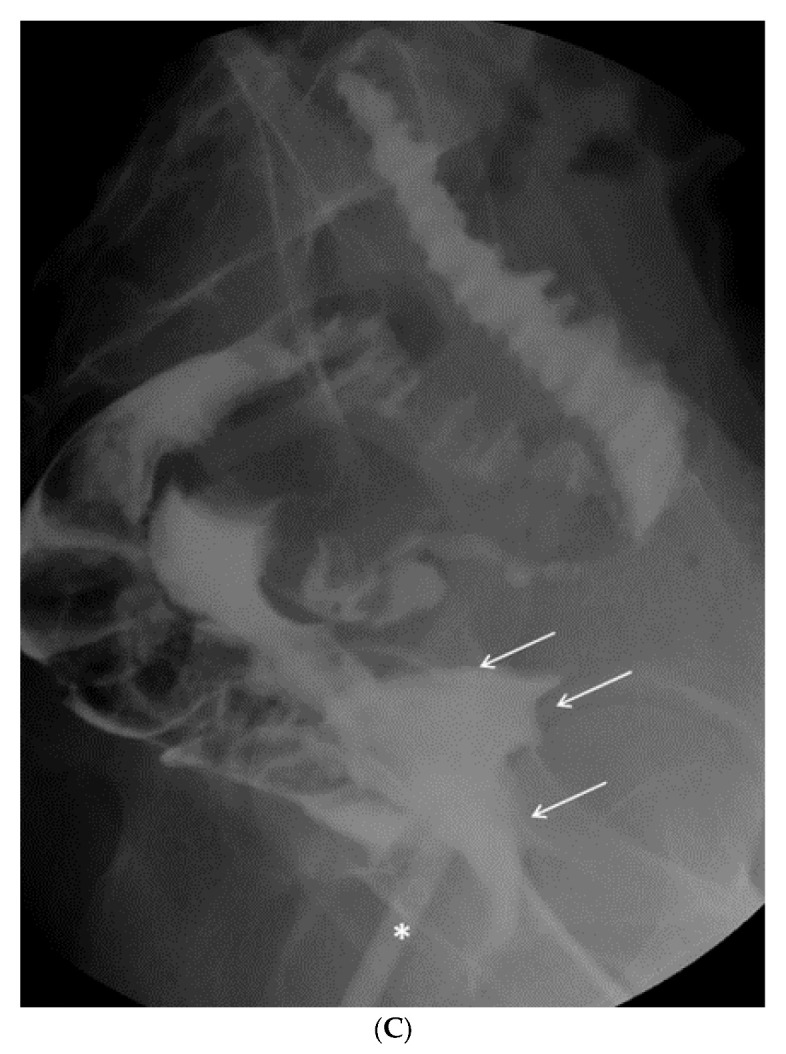
(**A**) Plain radiography shows free subdiaphragmatic air (white arrows). (**B**) Contrast-enhanced CT. A small fistula (white arrow) connects the rectum with the upper portion of the vaginal canal. Diffuse thickening of soft tissues close to the mesorectal fascia is associated with a thin fluid layer (asterisks). (**C**) A frame of fluoroscopy obtained through single-contrast enema performed with gastrografin. The contrast medium, injected transvaginally through a small tube (asterisk), spreads into the surrounding soft tissues (white arrows) and, to a lesser amount, into the rectum (arrowhead), confirming the presence of a pervious fistula.

The radiologic tests confirmed the clinical suspicion of vagino-rectal fistula (Figure 7A–C), and the patient underwent adequate surgical treatment.

After the diagnosis was made, surgical consultation was requested and, initially, the patient underwent diagnostic laparoscopy in which the presence of the fistula and a peritoneal picture of diffuse inflammation were noted. In view of the diffuse picture of initial peritonitis, a protective ostomy was packaged with the last ileal loop during surgery. Two drains were placed in the abdomen and antibiotic therapy started. The patient was discharged with antibiotic therapy and underwent outpatient follow-ups at the referring facility. About seven months later, a second operation was performed with detachment of the rectum from the recto-vaginal fistula and recto-vaginal septum and anterior resection of the rectum with colorectal T.T. anastomosis.

## 3. Results and Discussion

In the cervical cancer prevention pathway, colposcopists must have high-quality screening expertise. The American Society for Colposcopy and Cervical Pathology (ASCCP) has identified a number of quality indicators and proposals to improve the work of colposcopists. The doctor who performed the procedure is an experienced surgeon who has performed approximately two hundred conjectures [19].

The biopsy performed under colposcopic guidance confirmed the involvement of two-thirds of the entire thickness of the squamous epithelial lining, with a diagnosis of HSIL/CIN2.

Patients with a histological diagnosis of HSIL (CIN2-3) on biopsy examination should be treated. Patient observation alone is not considered an acceptable option [5,6], except in particular cases (pregnancy in the absence of colposcopic suspicion of invasion, young women < 25 years of age [20,21]).

Approximately two months later, the lesion progressed, and when the patient underwent conization with a loop diathermy, the cytological abnormalities involved the full thickness of the squamous epithelium, indicating HSIL/CIN3. Conization with loop diathermy offers numerous advantages and is currently the most-used excisional technique. The therapeutic success rate ranges from 90% to 97%. The excisional treatment is indicated to allow histological examination of the removed tissue, which is necessary to identify any tumors and plan the most appropriate oncological treatment. In fact, the frequency of “occult” invasive carcinomas can reach 12% of cases, primarily consisting of stage pT1A1, pT1A2, and stage 1B tumors. It should be noted that staging for stage 1A is postoperative [22].

Postoperative bleeding is usually minor, but in this case, bleeding from the uterine cervical branch necessitated the application of hemostatic sutures. The sutures were applied on the right vaginal wall, away from the rectum, excluding any transfixion involving the rectum and vagina. The thermal damage caused by the loop to the lateral walls of the cone is negligible (maximum thickness 50–300 μ), allowing the pathologist to make a satisfactory histological evaluation. In the case of extensive lesions, the excision site can be widened. Any bleeding originating from the walls due to the loss of substance created by the passage of the loop is then cauterized using a ball electrode, which also helps eliminate any glandular involvement [23,24,25].

According to Joule’s law, the amount of heat produced is directly proportional to the amount of current, its voltage, and the time it takes to pass through the tissue; it is inversely proportional to the conductivity of the tissue and the size of the object from which the current flows (i.e., the electrode). In the present case, the surgical time in which the current was used was similar to other conizations, not greater. In fact, the act of electroresection was not complicated, but the subsequent control of hemostasis was, in which no current was used but hemostatic sutures were. The electric current, suitably modulated by a modern generator, penetrates the tissues through an electrode (positive electrode) and exits the body picked up by a second electrode (negative electrode), generally in the form of a plate, rigid or flexible, applied at the shortest possible distance from the positive electrode, in order to minimize tissue damage to the surrounding tissues linked to the dispersion of the current, which, moreover, by generating heat, loses intensity. In this case, the position of the plate was corrected and the current was within the standard values.

The procedure was performed with the application of vaginal valves, and therefore, the cervix and vagina were examined before, during, and after the surgery, with no areas of vaginal mucosal injury observed. The integrity of the vaginal mucosa in its entirety and the proper removal of the pathological component of the cervix indicate the correct use of loop diathermy in all stages of the procedure, both in conization and subsequent widening. The CIN3 lesion, classified as carcinoma in situ in the international literature, was completely excised by widening the margins of the lesion. The presence of HR-HPV infection and CIN has been found to be associated with changes in the vaginal microbiota and alteration of local immune function, which may contribute to the pathogenesis of preterm delivery. These factors should also be considered in surgical procedures, as they can cause anatomical and functional damage to the cervix [26,27]. Inappropriate excisional treatment and subsequent difficult diagnosis due to poorly identifiable margins can mask an invasive lesion and, in cases of diagnostic uncertainty, lead to hysterectomy even in young women [28].

The most probable hypothesis is that the fistula was created due to secondary heat conduction; we assume that there was a pre-existing tissue alteration at the base, which could not be demonstrated. During conization, tissue of the cervix approximately 1.5 cm deep and 2 cm wide is removed in the vaginal component and thus away from other tissue. The extent of tissue damage is directly proportional to the temperature produced at a given point as a result of the passage of heat; in conization, the rectum is too far from the cervix for heat to pass into healthy tissue. The resulting cone excision is conical and contains a portion of the cervical canal; the thermal damage produced by the wire on the side walls of the cone is negligible (maximum thickness 50–300 μ) and presumably remains confined to the area of electroresection [29]. Early complications of conservative cervical interventions reported in the literature are hemorrhage and cervico-vaginal infection, of negligible entity (<1% of cases), often related to a lack of pre-operative vaginal disinfection or to incorrect post-operative patient behavior. In addition, electrosurgical damage in the transmission of inappropriate heat to residual healthy tissue may result in scarring and stenosis, but not fistulas. It has been shown that HR-HPV infection itself and the presence of CIN appear to be associated with changes in the vaginal microbiota and altered local immunological function that could lead to anatomo-functional damage of the cervix, including preterm delivery. The change in microbiota could be one of the contributing causes of altered tissue composition and resistance to heat transfer.

## 4. Conclusions

In conclusion, the clinical therapeutic pathway and subsequent surgical treatment can be considered appropriate. Recto-vaginal fistulas have not been reported in the literature as a complication of conization; electrosurgical damage in the transmission of inappropriate heat to residual healthy tissue may result in scarring and stenosis, but not fistulas. The lesion was present in all the enlargements, without which complete removal of the lesion would not have been achieved. The type of excision was modulated on the basis of the type of transformation zone and the size of the lesion, using a suitably sized loop, with the aim of sparing as much healthy tissue as possible, reducing the intracervical extension while maintaining therapeutic efficacy. The thermal damage is not considered a direct cause of the rectovaginal fistula, but it is likely an indirect cause due to HPV-related dysplasia associated with immune defense alteration, as well as due to dysentery, which can disrupt intestinal metabolism and lower its immune defenses, creating an inflammatory state that weakens tissue walls. The reason why, under immunocompetent conditions, HPV infection persists in some women rather than in others is not yet fully known [30] Certainly, a major role for clearance is age: infection with high-risk genotypes persists in more than 50% of women over 55 years of age, compared to 20% found in women under 25 years of age.

## Data Availability

Data are available from the corresponding author upon reasonable request.
